# Epidemiological profile of acute kidney injury in critically ill patients admitted to intensive care units: A Prospective Brazilian Cohort

**DOI:** 10.1590/2175-8239-JBN-2020-0191

**Published:** 2021-03-05

**Authors:** Antônio José Inda-Filho, Heitor Siqueira Ribeiro, Edilene Almeida Vieira, Aparecido Pimentel Ferreira

**Affiliations:** 1 Centro Universitário ICESP, Brasília, Distrito Federal, DF, Brasil.; 2 Universidade de Brasília, Brasília, Distrito Federal, DF, Brasil.

**Keywords:** Acute Kidney Injury, Intensive Care Units, Epidemiology, Nephrology, Lesão Renal Aguda, Unidades de Terapia Intensiva, Epidemiologia, Nefrologia

## Abstract

**Introduction:**

Acute kidney injury (AKI) is a frequent syndrome affecting patients admitted to intensive care units (ICU), and it is associated with poor clinical outcomes. The aim of the present study was to understand the epidemiological profile of patients with AKI admitted to ICUs.

**Methods:**

Prospective cohort study, carried out in three ICUs in the Federal District, Brazil. Between October/2017 and December/2018, 8,131 patients were included in the cohort. AKI was defined according to the KDIGO criteria. The main outcomes assessed were AKI development and mortality within 28 days of hospitalization.

**Results:**

Of the 8,131 patients followed up, 1,728 developed AKI (21.3%). Of the 1,728 patients with AKI, 1,060 (61.3%) developed stage 1, while stages 2 and 3 represented 154 (8.9%) and 514 (29.7%), respectively. Of these, 459 (26.6%) underwent renal replacement therapy. The mortality was 25.7% for those with AKI, and 4.9% for those without AKI.

**Discussion:**

Patients with AKI had higher mortality rates when compared to those without AKI. Likewise, among patients with AKI, higher disease stages were associated with higher death occurrences. AKI incidence (21.3%) and mortality (25.7%) in our study is in line with the largest meta-analysis ever conducted, in which incidence and mortality of 21.6 and 23.9% were observed, respectively. These findings confirm the importance of establishing the KDIGO guideline for the definition and management of AKI in Brazilian ICUs.

## Resumo

**Introdução:**

A injúria renal aguda (IRA) é uma síndrome frequente em pacientes admitidos em unidades de terapia intensiva (UTI) e está associada a negativos desfechos clínicos. O objetivo do presente estudo foi conhecer o perfil epidemiológico de pacientes com IRA admitidos em UTIs.

**Métodos:**

Estudo de coorte prospectiva, realizado em três UTIs do Distrito Federal, Brasil. Entre o período de outubro/2017 e dezembro/2018, 8.131 pacientes foram incluídos na coorte. A IRA foi definida de acordo com o critério KDIGO. Os principais desfechos avaliados foram o desenvolvimento de IRA e mortalidade dentro de 28 dias de internação.

**Resultados:**

Dos 8.131 pacientes acompanhados, 1.728 desenvolveram IRA (21,3%). Dos 1.728 pacientes com IRA, 1.060 (61,3%) desenvolveram o estágio 1, já os estágios 2 e 3 representaram 154 (8,9%) e 514 (29,7%), respectivamente. Destes, um total de 459 (26,6%) realizou terapia renal substitutiva. A mortalidade observada foi de 25,7% para aqueles com IRA e 4,9% para os não IRA.

**Discussão:**

Os pacientes com IRA, comparados aos não IRA, apresentaram maior mortalidade. Da mesma forma, entre os pacientes com IRA, os estágios superiores estiveram associados à maior ocorrência de óbito. A incidência de IRA (21,3%) e mortalidade (25,7%) em nosso estudo está em consonância com a maior meta-análise já conduzida, na qual foram observadas incidência e mortalidade de 21,6 e 23,9%, respectivamente. Esses achados confirmam a importância de se estabelecer a diretriz KDIGO para definição e manejo da IRA em UTIs brasileiras.

## Introduction

Acute kidney injury (AKI) is a frequent syndrome in patients admitted to intensive care units (ICU), and it is associated with increased length of hospital stay, high costs to the healthcare system and high mortality rates.^1^^-^ 3

Most epidemiological studies involving AKI have been carried out in North America, Europe and Asia; therefore, we have little information about it in Latin America.^4^^,^ 5 It seems that the outcomes perceived in AKI patients in developed countries are different when compared to those from developing countries.^6^ There are few studies in Brazil, with the few existing ones being retrospective in design or not bearing a very representative sample.^7^^,^ 8

A better understanding of the epidemiological profile of AKI in Brazil could open the field for a series of investigations, in order to better understand its relationship with length of hospital stay and mortality, in addition to enabling cost reductions in the healthcare system. In this sense, our goal with present study was to better understand the epidemiological profile of patients with AKI admitted to ICUs in the Federal District, Brazil.

## Methods

### Study Design

Observational prospective cohort study, carried out in critical patients admitted to three ICUs of tertiary hospitals in the Federal District during the period from October/2017 to December/2019. It was approved by the Research Ethics Committee of the Centro Universitário ICESP (nº 3.608.561). Eligible patients were those aged ≥18 years. Those receiving dialysis, with chronic kidney disease on renal replacement therapy (RRT), kidney transplant patients and with hospitalization <48 hours were excluded. We followed the STROBE recommendations (Strengthening the Reporting of Observational Studies in Epidemiology).^9^

### Data Collection

A previously trained team collected the data from the electronic records of the patients, and such team was led by a nurse specialized in clinical research (EAV). All data were collected at the time of admission to the ICU, daily. The collection included the baseline characteristics of the patients, comorbidities (hypertension, diabetes, heart failure, cancer, coronary artery disease, COPD, cirrhosis and others), clinical categories of ICU admission (pulmonary, sepsis, surgical, cardiovascular, gastrointestinal, infectious, neurological, orthopedic, oncological, acid-base and hydroelectrolytic disorders and others), serum creatinine and simplified acute physiological score (SAPS 3) to assess clinical severity and risk of mortality.

The following variables were investigated during follow up: development of AKI; need for RRT; use of mechanical ventilation; administration of vasoactive drugs or nephrotoxic antibiotics; length of stay in the hospital and ICU; and clinical outcome (death, discharge and hospital transference).

### Aki Definition And Stratification

All admitted and eligible patients were followed for 28 days in the ICUs, in another inpatient unit or until some outcome, such as hospital discharge, death or hospital transference. AKI was defined according to the criteria proposed by Kidney Disease: Improving Global Outcomes (KDIGO),^10^ of which: increase in serum creatinine ≥ 0.3 mg/dL within 48 hours or ≥ 50% in the first 7 days compared to creatinine levels upon admission to the ICU. We stratified the patients diagnosed with AKI according to the KDIGO: 1) increase in serum creatinine from 1.5 to 1.9 times or 0.3 mg/dL; 2) 2.0 to 2.9 times; and 3) ≥ 3.0 times the baseline value or serum creatinine ≥ 4.0 mg/dL or RRT onset. Urine output was not used for the diagnosis and stratification of AKI, because of the difficulties presented in the collection and appointment by the ICU technical personnel.

### Statistical Analysis

Descriptive statistics was conducted using the mean, standard deviation and frequency values. Kolmogorov-Smirnov test was used to check data normality. To compare continuous variables between groups with and without AKI, we used the independent Student’s t-test. For categorical variables and frequencies, the chi-square test was applied. The level of significance was set at 95%. The analysis was perfomed using the Statistical Package for the Social Sciences (SPSS) program, version 26.0 (SPSS Inc., Chicago, IL, USA).

## Results

A total of 8,131 patients were admitted and followed for a period of 9.3 ± 16.1 days in the ICUs. Table 1 shows the admission characteristics of the patients included in the cohort.

**Table 1:** Sample admission characteristics (n=8131)

Variables	Total (n=8131)	AKI (n=1728)	Non-AKI (n=6403)	p
Number of patients	8131 (100%)	1728 (21.3%)	6403 (78.7%)	<0.001
Age (years)	66.0±19.0	70.4±16.3	64.9±19.5	<0.001
Females	4254 (52.3%)	855 (49.5%)	3399 (53.1%)	0.008
**Comorbidities**				
Hypertension	4363 (53.7%)	1111 (64.3%)	3252 (50.8%)	<0.001
Diabetes	2272 (27.9%)	604 (35.0%)	1668 (26.1%)	<0.001
Heart failure	501 (6.2%)	234 (12.2%)	328 (4.9%)	<0.001
Cancer	937 (11.5%)	207 (12.0%)	730 (11.4%)	0.504
Coronary artery disease	830 (10.2%)	225 (13.0%)	605 (9.4%)	<0.001
COPD	385 (4.7%)	97 (5.6%)	288 (4.5%)	0.053
Cirrhosis	81 (1.0%)	19 (1.1%)	62 (1.0%)	0.626
**ICU admission categories**				
Pneumological	1229 (15.1%)	253 (15.1%)	968 (15.1%)	0.989
Sepsis	829 (10.2%)	224 (13.0%)	605 (9.4%)	<0.001
Surgical	1783 (20.6%)	223 (12.9%)	878 (13.7%)	0.384
Cardiovascular	1176 (14.5%)	352 (20.4%)	824 (12.9%)	<0.001
Gastrointestinal	282 (7.2%)	97 (5.6%)	485 (7.6%)	0.005
Infection	427 (5.3%)	50 (2.9%)	377 (5.9%)	<0.001
Neurological	1113 (13.7%)	215 (12.4%)	898 (14.0%)	0.089
Orthopedic	130 (1.6%)	21 (1.2%)	109 (1.7%)	0.152
Oncologic	53 (0.7%)	11 (0.6%)	42 (0.7%)	0.929
ABFD	153 (1.9%)	21 (1.2%)	132 (2.1%)	0.022
Others	1338 (16.5%)	253 (14.6%)	1085 (16.9%)	0.022
Creatinine upon admission (mg/dL)	1.16±1.03	1.55±1.76	1.05±0.68	<0.001
SAPS 3 score	18.6±17.4	25.8±20.8	16.7±15.8	<0.001

ABFD = Acid-base and fluid disorders; COPD = chronic obstructive pulmonary disease; SAPS 3 = simplified acute physiological score; AKI = acute kidney injury; ICU = Intensive Care Unit.

The number of patients who developed AKI in the first 7 days of hospitalization was 1,728 (21.3%). The creatinine level of patients upon admission who developed AKI was 1.55 ± 1.76 mg/dL; higher when compared to those who did not develop AKI. There were two or more comorbidities in 35.2% of the admitted patients. According to SAPS 3, patients who had AKI had a higher predictive mortality rate than those without AKI (25.8 ± 20.8 vs. 16.7 ± 15.8; <0.001).

Table 2 shows the variables monitored and clinical outcomes during the ICU stay.

**Table 2:** Variables followed and clinical outcomes during hospital stay (n=8131)

Variables	Total (n=8131)	AKI (n=1728)	Non-AKI (n=6403)	p
Renal replacement therapy	-	459 (26.6%)	-	-
**AKI stages**				
AKI stage 1	-	1060 (61,3%)	-	-
AKI stage 2	-	154 (8,9%)	-	-
AKI stage 3	-	514 (29.7%)	-	-
**Clinical outcome**				
Death	755 (9.3%)	444 (25.7%)	311 (4.9%)	<0.001
Discharge	7314 (90.0%)	1263 (73.1%)	6051 (94.5%)	<0.001
Transference	62 (0.7%)	21 (1.2%)	41 (0.6%)	
Nephrotoxic antibiotic	4108 (50.5%)	1047 (60.6%)	3061 (47.8%)	<0.001
Vasoactive drug	570 (7.0%)	294 (17.0%)	276 (4.3%)	<0.001
Mechanical ventilation	897 (11.0%)	480 (27.8%)	417 (6.5%)	<0.001
ICU stay (days)	9.3±16.1	14.5±23.2	7.9±13.3	<0.001
Hospital stay (days)	12.0±16.4	15.8±19.1	11.1±15.6	<0.001

AKI = acute kidney injury; ICU = intensive care unit.

The patients who developed AKI had a higher prevalence of arterial hypertension (64.3%) and diabetes (35%), as well as greater use of vasoactive drugs (17.0% vs. 4.3%; p <0.001) and mechanical ventilation (27.8% vs. 6.5%; p <0.001). The length of ICU stay was longer for patients who developed AKI (14.5 ± 23.2 vs. 7.9 ± 13.3; p <0.001) and the hospital stay was 15.8 ± 19.1 vs. 11.1 ± 15.6; p <0.001.

Etiologically, 224 patients with AKI had sepsis (13.0%). Another 253 patients (15.1%) had a pneumological cause, with pneumonia being the most prevalent. In addition, 1,047 of the patients with AKI (60.6%) used nephrotoxic antibiotics.

**Figure 1:**
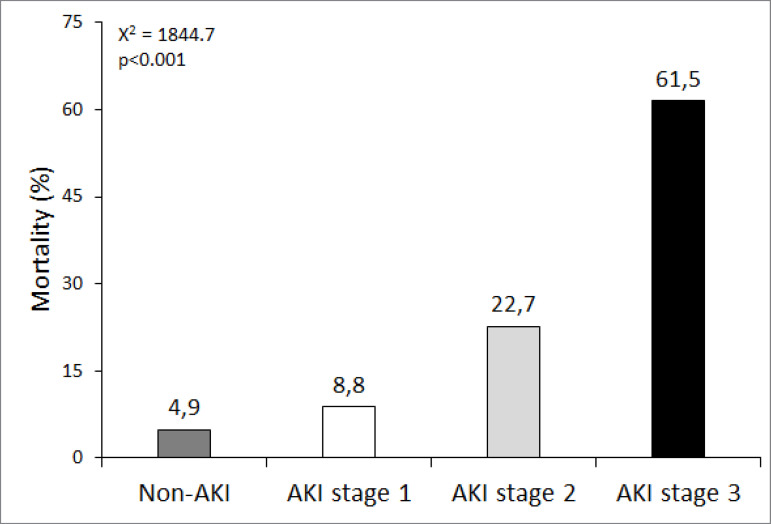
Mortality according to the Acute Kidney Injury.

Of the 1,728 patients with AKI, 1,060 (61.3%) developed stage 1, while stages 2 and 3 represented 154 (8.9%) and 514 (29.7%), respectively. A total of 459 (26.6%) underwent RRT, with the conventional modality being the most used (61.2%). In Figure 1, it is possible to identify mortality according to the stages of AKI.

Mortality increased as the stage of AKI increased. Mortality was significantly higher in those patients with AKI versus those without AKI (p <0.001).

## Discussion

The findings of the present study demonstrate the clinical severity of critically ill patients admitted to ICUs who develop AKI; these patients were older, had a higher number of comorbidities and had sepsis and cardiovascular pathology as the main admission categories. In addition, patients with AKI spent more time in the hospital and in the ICU, used more nephrotoxic antibiotics and vasoactive drugs, as well as required mechanical ventilation, demonstrating greater severity when compared to those patients who did not develop AKI. It is important to notice that, among patients who developed AKI, more than 60% were using nephrotoxic antibiotics. Despite all the technological advances, hospital-acquired infection remains a cause of high rates of morbidity and mortality worldwide and, unfortunately, bacterial resistance resulting from the injudicious use of antibiotics is a serious problem. In this study we did not perform a specific analysis to investigate the reasons for the high use of these antibiotics and the impact of AKI development, which will be better explored in future publications.

Finally, confirming our hypothesis, patients with AKI had higher mortality, and, similar to Hoste et al^11^ there was a gradual increase in mortality, with an increase in severity in AKI stages. AKI is a frequent and important problem in critically-ill patients, and its prevalence has increased considerably, both in developed and developing countries, to the point of becoming a public healthcare problem.^3^^,^ 12 Most published epidemiological studies are from developed countries, but represent only 15% of the world population.^4^ The worldwide incidence of AKI was analyzed in a meta-analysis with 312 studies. Of these, 154 (130 in adults and 24 in children) totaled more than 3.5 million patients, and the incidence of AKI by KDIGO criteria was 23.2%. In the other 158 studies, they used different definition criteria for AKI. South America had 11 studies included, all Brazilian: 8 retrospective, 2 prospective, 1 post-hoc, and only 6 adopted the KDIGO definition.^4^

The KDIGO definition for AKI diagnosis has been adopted as a consensus established by experts worldwide.^4^^,^ 13 Despite this, many Brazilian intensive care physicians continue to adopt other guidelines.^13^ The incidence of AKI (21.3%) and mortality (25.7 %) in our study is in line with the meta-analysis conducted by the Acute Kidney Injury Advisory Group of the American Society of Nephrology, 4 with incidence and mortality of 21.6 and 23.9%, respectively. These findings confirm the importance of establishing the KDIGO guideline for the definition and management of AKI in Brazilian ICUs.

Based on our survey conducted in the literature, this is the largest prospective cohort study in Latin America to assess AKI occurrence in ICUs using the KDIGO criteria for diagnosis and classification. In the Federal District, where the sample was collected, there is a high concentration of inhabitants from other states and other regions of Brazil; therefore, we suggest that these findings may represent the reality of Brazil, especially in private ICUs; however, this may be different in public ICUs.

Our study has some characteristics that show a certain robustness in our findings, such as: i) large sample size; ii) prospective methodological design; therefore, we monitor the admitted patients daily, unlike the vast majority of cohorts, which have a retrospective design; and iii) large participation of inhabitants from other regions of Brazil, which gives the study a possible portrait of the epidemiological profile of AKI in private ICUs in Brazil.

The main limitations are: i) the fact that data collection occurred only in patients in ICUs, not representing the AKI acquired in the community. Another limitation was the fact that only three private hospitals were involved in the study, although it represents different regions of the Federal District. Finally, it was not possible to obtain serum creatinine results from at least the last three months prior to admission to the ICU, which led us to evaluate the serum creatinine upon admission to the ICU to monitor possible AKI occurrence.

We concluded, through our cohort, that the incidence of AKI was 21.3%. There was a higher mortality of patients with AKI, compared to those who did not develop it. Likewise, among patients with AKI, higher disease stages were associated with higher mortality rates.

## References

[1] Hoste EAJ, Kellum JA, Selby NM, Zarbock A, Pavalevsky PM, Bagshaw SM (2018). Global epidemiology and outcomes of acute kidney injury. Nat Rev Nephrol.

[2] Singbartl K, Kellum JA (2012). AKI in the ICU: definition, epidemiology, risk stratification, and outcomes. Kidney Int.

[3] Santos RP, Carvalho ARS, Peres LAB, Ronco C, Macedo E (2019). An epidemiologic overview of acute kidney injury in intensive care units. Rev Assoc Med Bras.

[4] Susantitaphong P, Cruz DN, Cerda J, Abulfaraj M, Alqahtani F, Koulouridis I (2013). World incidence of AKI: A meta-analysis. Clin J Am Soc Nephrol.

[5] Lombardi R, Yu L, Younes-Ibrahim M, Schor N, Burdmann EA (2008). Epidemiology of acute kidney injury in Latin America. Semin Nephrol.

[6] Bouchard J, Acharya A, Cerda J, Maccariello ER, Madarasu RC, Tolwani AJ (2015). A prospective international multicenter study of AKI in the intensive care unit. Clin J Am Soc Nephrol.

[7] Ponce D, Zamoner W, Batistoco MM, Balbi A (2020). Changing epidemiology and outcomes of acute kidney injury in Brazilian patients: a retrospective study from a teaching hospital. Int Urol Nephrol.

[8] Santos RP, Carvalho ARS, Peres LAB (2019). Incidence and risk factors of acute kidney injury in critically ill patients from a single centre in Brazil: a retrospective cohort analysis. Sci Rep.

[9] Von Elm E, Altman DG, Egger M, Pocock SJ, Gotzsche PC, Vandenbroucke JP (2007). Strengthening the reporting of observational studies in epidemiology (STROBE) statement: guidelines for reporting observational studies. BMJ.

[10] Kidney Disease: Improving Global Outcomes (2012). KDIGO clinical practice guideline for acute kidney injury. Kidney Int Suppl.

[11] Hoste EAJ, Bagshaw SM, Bellomo R, Cely CM, Colamn R, Cruz DN (2015). Epidemiology of acute kidney injury in critically ill patients: the multinational AKI-EPI study. Intensive Care Med.

[12] Li PKT, Burdmann EA, Mehta RL, World Kidney Day Steering Committee 2013 (2013). Acute kidney injury: global health alert. Kidney Int.

[13] Ostermann M, Bellomo R, Burdmann EA, Doi K, Endre ZH, Goldstein SL (2020). Controversies in acute kidney injury: conclusions from a Kidney Disease: Improving Global Outcomes (KDIGO) Conference. Kidney Int.

